# Complete mitochondrial genomes of three skippers in the tribe Aeromachini (Lepidoptera: Hesperiidae: Hesperiinae) and their phylogenetic implications

**DOI:** 10.1002/ece3.7666

**Published:** 2021-05-18

**Authors:** Xiangyu Hao, Jiaqi Liu, Hideyuki Chiba, Jintian Xiao, Xiangqun Yuan

**Affiliations:** ^1^ College of Life Sciences Northwest A&F University Yangling China; ^2^ Key Laboratory of Plant Protection Resources and Pest Management Ministry of Education Entomological Museum College of Plant Protection Northwest A&F University Yangling China; ^3^ B.P. Bishop Museum Honolulu HI USA

**Keywords:** mitochondrial DNA, mitogenome, phylogeny

## Abstract

The mitochondrial genome is now widely used in the study of phylogenetics and molecular evolution due to its maternal inheritance, fast evolutionary rate, and highly conserved gene content. To explore the phylogenetic relationships of the tribe Aeromachini within the subfamily Hesperiinae at the mitochondrial genomic level, we sequenced and annotated the complete mitogenomes of 3 skippers: *Ampittia virgata*, *Halpe nephele,* and *Onryza maga* (new mitogenomes for 2 genera) with a total length of 15,333 bp, 15,291 bp, and 15,381 bp, respectively. The mitogenomes all contain 13 protein‐coding genes (PCGs), 22 transfer RNAs (tRNAs), 2 ribosomal RNAs (rRNAs), and a noncoding A + T‐rich region and are consistent with other lepidopterans in gene order and type. In addition, we reconstructed the phylogenetic trees of Hesperiinae using maximum likelihood (ML) and Bayesian inference (BI) methods based on mitogenomic data. Results show that the tribe Aeromachini in this study robustly constitute a monophyletic group in the subfamily Hesperiinae, with the relationships Coeliadinae + (Euschemoninae + (Pyrginae + ((Eudaminae + Tagiadinae) + (Heteropterinae + ((Trapezitinae + Barcinae) + Hesperiinae))))). Moreover, our study supports the view that *Apostictopterus fuliginosus* and *Barca bicolor* should be placed out of the subfamily Hesperiinae.

## Graphical abstract

To explore the phylogenetic relationships of the tribe Aeromachini within the subfamily Hesperiinae at the mitochondrial genomics level, we sequenced and annotated the complete mitogenomes of three skippers: *Ampittia virgata*, *Halpe nephele*, and *Onryza maga*. Results show that the 3 Aeromachini species in this study robustly constitute a monophyletic group in the subfamily Hesperiinae, with the relationships Coeliadinae + (Euschemoninae + (Pyrginae + ((Eudaminae + Tagiadinae) + (Heteropterinae + ((Trapezitinae + Barcinae) + Hesperiinae))))). Moreover, our study supports the view that *Apostictopterus fuliginosus* and *Barca bicolor* should be placed out of the subfamily Hesperiinae.

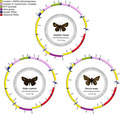

## 1 INTRODUCTION

The family Hesperiidae (skippers) is one of the most speciose families in the butterflies and consists of about 567 genera and more than 4,000 species around the world (Warren et al., 2008), accounting for one‐fifth of the world's butterfly species, though the number is far underestimated. The higher classification of the family had mainly followed Evans (Evans, 1943, 1949, 1951) until Warren et al. inferred the phylogenetic relationship from molecular (three loci) and morphological data of 196 genera (Warren et al., 2008, 2009). The latest molecular study of 250 hesperiid species from all over the world (Li, Cong, et al., 2019) and its supplementary study (Zhang et al., 2019) showed that the family Hesperiidae should be classified into 12 subfamilies, based on the timing of divergence, with the relationship of (Coeliadinae + (Euschemoninae + ((Eudaminae + (Tagiadinae + (Pyrrhopyginae +Pyrginae))) + (Katreinae + (Chamundinae + (Heteropterinae + (Barcinae +Trapezitinae) + Hesperiinae)))))), but this higher classification and the phylogeny has not been approved generally. Hesperiinae, the largest subfamily, has been acknowledged as a distinctly monophyletic group as monophyletic in the hypothesis of Li, Cong, et al. (2019), Sahoo et al. (2016), Toussaint et al. (2018), Warren et al. (2009), and Zhang et al. (2019). There are 11 mitochondrial genomes of Hesperiinae directly available on GenBank, and the rest can be accessed in the form of raw data. We have provided more comprehensive data support for the phylogenetic research of the groups.

Aeromachini is a large and diverse tribe of the subfamily Hesperiinae and currently contains approximately 130 species in 12 genera, distributed in the Oriental Region, the Palearctic Region, and the Afrotropical Region (Cock & Congdon, 2012; Devyatkin, 1996; Evans, 1949; Huang et al., 2019; Warren et al., 2009; Yuan et al., 2015). Most of the genera of Aeromachini are distributed in the Sino‐Himalayan Subregion. In the previous phylogenetic studies, the tribe is always retrieved as a clade sister to the rest of the Hesperiinae (Li, Cong, et al., 2019). Two molecular studies within the tribe are known (Zhang et al., 2019; Li, Zhu, et al., 2019).

The mitogenome is the most extensively studied genomic system in insects, which is a double strand molecule about 15~16 kb in size, typically containing 13 protein‐coding genes (PCGs), 22 transfer RNAs (tRNAs), 2 ribosomal RNAs (rRNAs), and a noncoding A + T‐rich region. In the past few decades, due to its maternal inheritance, fast evolutionary rate and highly conserved gene content compared to nuclear genes, it has been widely utilized to investigate insect taxonomy, phylogenetic relationships, evolution, and biogeography, as a source of sequence data for phylogenetic analysis. (Cameron, 2014; Galtier et al., 2009). In this study, we determined the complete mitochondrial genome sequences of 3 skipper species of the tribe Aeromachini and reconstructed the phylogenetic relationships of the family Hesperiidae, combined with other available sequence data in GenBank, and using maximum likelihood and Bayesian inference methods, aiming to provide new horizons and genomics data for the phylogenetic research of the Aeromachini.

## 2 MATERIALS AND METHODS

### 2.1 Sample collection and DNA extraction

Adult specimens of *Ampittia virgate* Leech, 1890 and *Halpe nephele* Leech, 1893 were sampled at Jiuxian Mountain and Wuyi Mountain in Fujian Province, China, respectively, in July 2016. The adult *Onryza maga* Leech, 1890 was collected at Matou Mountain, Jiangxi Province, China in August 2018. All materials were preserved in 100% ethanol immediately after collecting and stored at −20˚C at the Entomological Museum of the Northwest A&F University, Yangling, Shaanxi Province, China. Specimen identification was based on morphological characteristics following Yuan et al. (2015) and the identity was confirmed via *cox1* barcoding using the BOLD database (Ratnasingham & Hebert, 2013). The genomic DNA was isolated from the thoracic tissue using the EasyPure^R^ Genomic DNA Kit (TransGen Biotech, Beijing).

### 2.2 Sequencing, assembly, annotation, and bioinformatic analyses

Three complete mitogenomes were sequenced using next‐generation sequencing (NGS) on an Illumina HiSeq 2000 platform (Biomarker Technologies, Beijing). The correct recognition rate of bases reaches 99.9%. Each Illumina HiSeq read was 150 bp, and about 1.2 Gb raw data were trimmed with default parameters, then the raw paired reads were retrieved and quality‐trimmed using CLC Genomics Workbench v10.0.1 (CLC Bio, Aarhus, Denmark) with default parameters. The clean paired reads were then used for mitogenome reconstruction using MITObim v1.7 software (Hahn et al., 2013) with default parameters and the mitogenome of *Ampittia dioscorides* (KM102732) (Qin et al., 2017) as the reference. Annotation of the mitogenomes and comparative analyses were conducted following the methodology outlined above. The various genomic features were annotated using Geneious 8.1.3 (Biomatters, Auckland, New Zealand) and referenced to the complete mitogenome sequence of *A*. *dioscorides*. Protein‐coding genes (PCGs) were determined by finding the ORFs based on the invertebrate codon table (codon Table 5), and RNAs (tRNAs and rRNAs) were identified using MITOS Web Server (Bernt et al., 2013). Transfer RNAs were manually plotted according to the secondary structure predicted by MITOS, using Adobe Illustrator CS5. Finally, all genes were visually inspected against the reference mitogenome in Geneious. Nucleotide composition, codon usage, comparative mitogenomic architecture tables for the three mitogenomes, and data used to plot RSCU (relative synonymous codon usage) figures were all calculated and created using PhyloSuite (Zhang et al., 2020). The AT‐skew and GC‐skew were computed according to the following formulas: AT‐skew = [A − T]/[A + T] and GC‐skew = [G − C]/[G + C] (Perna & Kocher, 1995). The three newly sequenced mitogenome sequences of Aeromachini (*Ampittia virgata*, *Halpe nephele,* and *Onryza maga*) have been uploaded onto GenBank with the accession number MW288057, MW288058, and MW288059, respectively.

### 2.3 Sequence read archive (SRA) data extraction

We referred to and used the data of six genomes from over 300 hesperiid species determined in previous study to extract the mitochondrial genomes because of the lack of directly available mitogenomes on GenBank. The SRA data of the subfamily Trapezitinae (*Hewitsoniella migonitis*, *Anisynta dominula*, *Toxidia parvulus,* and *Signeta flammeata*) and two species of the tribe Aeromachini (*Aeromachus stigmata* and *Ampittia dioscorides*) were obtained from GenBank with the DNA Voucher NVG‐17108D07, NVG‐17069D05, NVG‐7813, NVG‐7760, NVG‐7915, and NVG‐7291, respectively (Li, Cong, et al., 2019; Zhang et al., 2019). The raw data of 4 species of Trapezitinae were assembled into mitogenomes referred to *Barca bicolor* (Han et al., 2018), and the 2 species of Aeromachini were referred to *Isoteinon lamprospilus* (Ma et al., 2020) using Geneious.

### 2.4 Phylogenetic analysis

A total of 41 species (3 newly determined in this study, 38 available from GenBank) representing 9 subfamilies of Hesperiidae sens Li, Cong, et al. (2019) and Zhang et al. (2019) were used to reconstruct their phylogenetic relationships. The ingroup contains 5 species of Coeliadinae, 1 species of Euschemoninae, 2 species of Pyrginae, 4 species of Tagiadinae, 2 species of Eudaminae, 3 species of Heteropterinae, 2 species of Barcinae, 4 species of Trapezitinae, and 18 species of Hesperiinae. The 4 Papilionidae species (*P*. *machaon*, *P*. *helenus*, *G*. *timur* and *P*. *apollo*) were selected as outgroups (Table 1).

**TABLE 1:** The mitochondrial genome sequences of the 35 Hesperiidae species and 4 Papilionidae outgroup species used in this study

Taxon	Species	Accession number/DNA Voucher	References
Hesperiidae			
Coeliadinae	*Burara striata*	NC_034676	Zhang, Cong, Shen, Wang, et al. (2017)
	*Choaspes benjaminii*	NC_024647	Kim et al. (2014)
	*Hasora anura*	KF881049	Wang et al. (2016)
	*Hasora vitta*	NC_027170	Cao et al. (2016)
	*Hasora badra*	NC_045249	Unpublished
Euschemoninae	*Euschemon rafflesia*	NC_034231	Zhang, Cong, Shen, Fan, et al. (2017)
Tagiadinae	*Celaenorrhinus maculosus*	NC_022853	Wang et al. (2015)
	*Ctenoptilum vasava*	JF713818	Hao et al. (2012)
	*Daimio tethys*	KJ813807	Zuo et al. (2016)
	*Tagiades vajuna*	KX865091	Liu et al. (2017)
Pyrginae	*Pyrgus maculatus*	NC_030192	Unpublished
	*Erynnis montanus*	NC_021427	Wang et al. (2014)
Eudaminae	*Achalarus lyciades*	NC_030602	Shen et al. (2016)
	*Lobocla bifasciata*	KJ629166	Kim et al. (2014)
Heteropterinae	*Carterocephalus silvicola*	NC_024646	Kim et al. (2014)
	*Heteropterus morpheus*	NC_028506	Unpublished
	*Leptalina unicolour*	MK265705	Jeong et al. (2019)
Barcinae	*Apostictopterus fuliginosus*	NC_039946	Han et al. (2018)
	*Barca bicolor*	NC_039947	Han et al. (2018)
Trapezitinae	*Hewitsoniella migonitis*	NVG‐17108D07	Li, Cong, et al. (2019)
	*Anisynta dominula*	NVG‐17069D05	Li, Cong, et al. (2019)
	*Toxidia parvulus*	NVG‐7813	Li, Cong, et al. (2019)
	*Signeta flammeata*	NVG‐7760	Li, Cong, et al. (2019)
Hesperiinae	*Ampittia virgata*	MW288057	This study
	*Halpe nephele*	MW288058	This study
	*Onryza maga*	MW288059	This study
	*Lerema accius*	NC_029826	Cong and Grishin (2016)
	*Ochlodes venata*	HM243593	Unpublished
	*Parnara guttata*	NC_029136	Shao et al. (2015)
	*Potanthus flavus*	KJ629167	Kim et al. (2014)
	*Astictopterus jama*	MH763663	Ma et al. (2020)
	*Isoteinon lamprospilus*	MH763664	Ma et al. (2020)
	*Notocrypta curvifascia*	MH763665	Ma et al. (2020)
	*Agathymus mariae*	KY630504	Shen et al. (2017)
	*Megathymus beulahae*	KY630505	Zhang, Cong, et al. (2017)
	*Megathymus cofaqui*	KY630503	Zhang, Cong, et al. (2017)
	*Megathymus streckeri*	KY630501	Zhang, Cong, et al. (2017)
	*Megathymus ursus*	KY630502	Zhang, Cong, et al. (2017)
	*Megathymus yuccae*	KY630500	Zhang, Cong, et al. (2017)
	*Aeromachus stigmata*	NVG‐7915	Li, Cong, et al. (2019)
	*Ampittia dioscorides*	NVG‐7291	Li, Cong, et al. (2019)
Outgroup			
Papilionidae	*Papilio machaon*	NC_018047	Unpublished
	*Papilio helenus*	NC_025757	Tang et al. (2014)
	*Graphium timur*	NC_024098	Chen et al. (2016)
	*Parnassius apollo*	NC_024727	Chen et al. (2014)

The complete mitogenome genes were extracted using PhyloSuite v1.2.2, and the sequences of 13 PCGs of the 39 species were aligned in batches with MAFFT integrated into PhyloSuite. Nucleotide sequences were aligned using the G‐INS‐i (accurate) strategy and codon alignment mode. All rRNAs were aligned in the MAFFT with the Q‐INS‐i strategy (Katoh & Standley, 2013). Poorly matched sites in the alignments were removed using Gblocks v0.91b (Castresana, 2000). Individual genes were also concatenated using PhyloSuite v1.2.2.

We used 3 datasets to reconstruct the phylogenetic relationship: (1) PCG matrix, containing all codon positions of the 13 protein‐coding genes; (2) PRT matrix, concatenating all codon positions of the 13 protein‐coding genes, 22 tRNAs and 2 rRNAs; and (3) 12PRT matrix, including the first and second codon positions of 13 protein‐coding genes plus 22 tRNAs and 2 rRNAs. Based on 3 datasets, the maximum likelihood (ML) and Bayesian inference (BI) methods were used to reconstruct the phylogeny. The optimal partitioning scheme and nucleotide substitution model for ML and BI phylogenetic analyses were selected using PartitionFinder 2.1.1 (Lanfear et al., 2017) with the greedy algorithm and BIC (Bayesian information criterion) criteria (Tables S1 and S2). Maximum likelihood analysis was inferred using IQ‐TREE (Nguyen et al., 2015) with the standard bootstrap approximation approach, as well as the Shimodaira–Hasegawa‐like approximate likelihood ratio test (Guindon et al., 2010), and the bootstrap value (BS) of each node of the ML tree was evaluated via the bootstrap test with 10,000 replicates. Bayesian inference was carried out using MrBayes 3.2.6 (Ronquist et al., 2012) with the following requirements: 2 independent runs of 1 × 10^7^ generations were conducted with four independent Markov Chain Monte Carlo (MCMC) runs, including 3 heated chains and a cold chain, by sampling every 1,000 generations. A consensus tree was obtained from all the trees after the initial 25% of trees from each MCMC run was discarded as burn‐in, with the chain convergence assumed after the average standard deviation of split frequencies fell below 0.01. The confidence value of each node of the BI tree was presented as the Bayesian posterior probability (BP).

## 3 RESULTS AND DISCUSSION

### 3.1 Mitogenome organization and base composition

**FIGURE 1:**
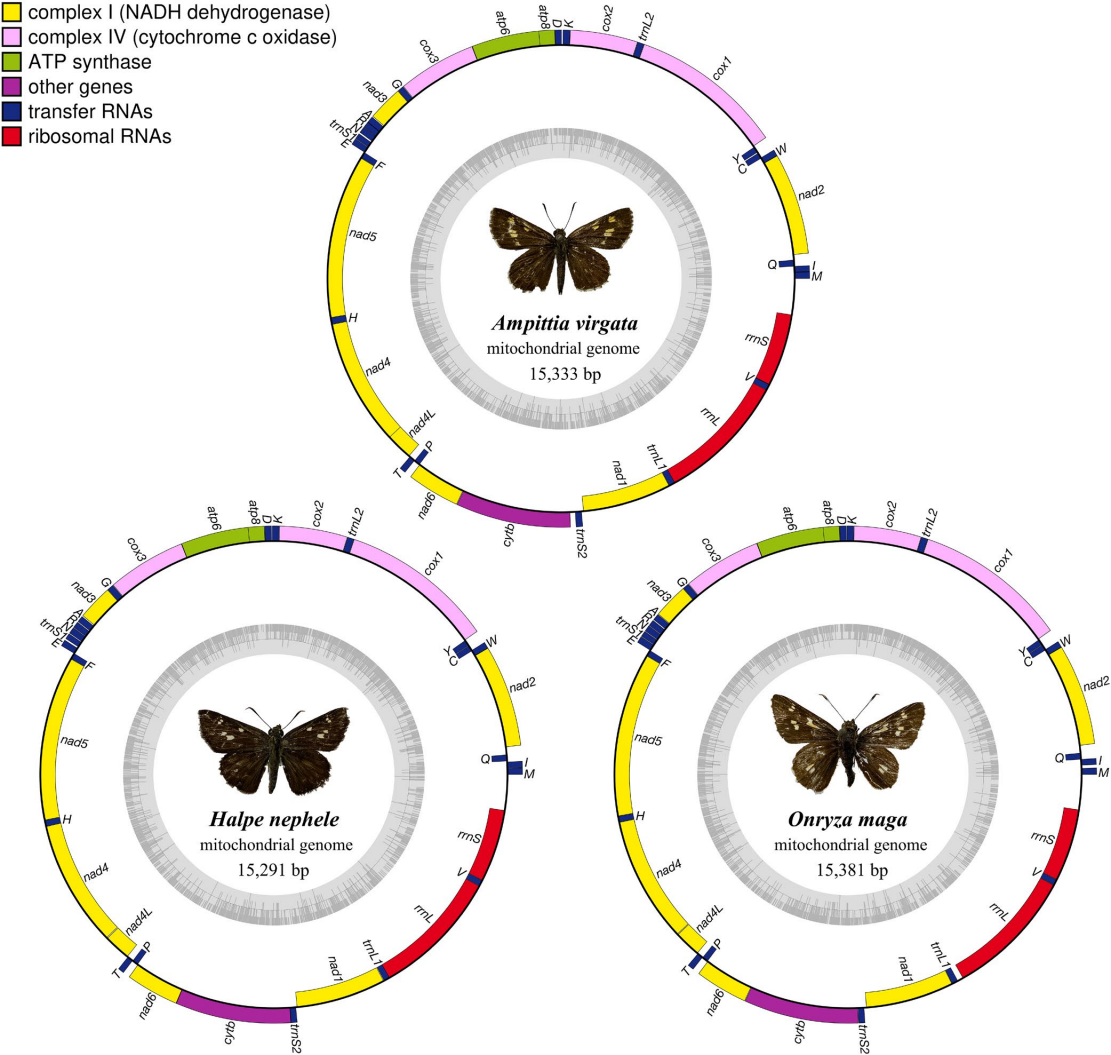
Circular maps of the mitochondrial genomes of *Ampittia virgata*, *Halpe nephele*, and *Onryza maga*

The total lengths of the mitogenomes of *Ampittia virgata*, *Halpe nephele,* and *Onryza maga* are 15,333 bp, 15,291 bp, and 15,381 bp, respectively (Figure 1). The gene order and organization are similar to those of other butterflies previously determined, containing 13 protein‐coding genes (PCGs), 22 transfer RNAs (tRNAs), 2 ribosomal RNAs (rRNAs), and a noncoding A + T‐rich region. Among them, 14 genes are encoded from the N‐strand, and the remaining 23 genes are from the J‐strand (Table 2).

**TABLE 2:** Mitogenomic organization of *Ampittia virgata*, *Halpe nephele*, and *Onryza maga*

Gene	Position		Size	Intergenic nucleotides	Codon		Strand
	From	To			Start	Stop	
*A. virgata*/*H. nephele*/*O. maga*							
*trnM*	1/1/1	67/68/68	67/68/68				J/J/J
*trnI*	67/69/99	130/133/162	64/65/64	−1/–/30			J/J/J
*trnQ*	128/141/160	196/209/228	69/69/69	−3/7/−3			N/N/N
*nad2*	250/288/306	1263/1301/1319	1014/1014/1014	53/78/77	ATT/ATT/ATT	TAA/TAA/TAA	J/J/J
*trnW*	1262/1300/1318	1328/1366/1384	67/67/67	−2/−2/−2			J/J/J
*trnC*	1321/1359/1377	1385/1423/1441	65/65/65	−8/−8/−8			N/N/N
*trnY*	1395/1425/1443	1460/1490/1507	66/66/65	9/1/1			N/N/N
*cox1*	1471/1493/1514	3001/3023/3044	1531/1531/1531	10/2/6	CGA/CGA/CGA	T/T/T	J/J/J
*trnL2*	3002/3024/3045	3068/3090/3111	67/67/67				J/J/J
*cox2*	3069/3091/3112	3744/3769/3787	676/679/676		ATG/ATG/ATG	T/T/T	J/J/J
*trnK*	3745/3770/3788	3815/3840/3858	71/71/71				J/J/J
*trnD*	3827/3845/3861	3893/3914/3929	67/70/69	11/4/2			J/J/J
*atp8*	3894/3915/3930	4055/4079/4094	162/165/165		ATA/ATT/ATT	TAA/TAA/TAA	J/J/J
*atp6*	4049/4073/4088	4726/4750/4765	678/678/678	−7/−7/−7	ATG/ATG/ATG	TAA/TAA/TAA	J/J/J
*cox3*	4726/4750/4765	5511/5535/5550	786/786/786	−1/−1/−1	ATG/ATG/ATG	TAA/TAA/TAA	J/J/J
*trnG*	5514/5538/5553	5577/5604/5618	64/67/66	2/2/2			J/J/J
*nad3*	5578/5605/5619	5931/5958/5972	354/354/354		ATT/ATT/ATT	TAA/TAA/TAA	J/J/J
*trnA*	5939/5967/5976	6005/6032/6043	67/66/68	7/8/3			J/J/J
*trnR*	6005/6038/6051	6067/6103/6115	63/66/65	−1/5/7			J/J/J
*trnN*	6068/6106/6118	6133/6172/6182	66/67/65	–/2/2			J/J/J
*trnS1*	6147/6178/6186	6207/6238/6246	61/61/61	13/5/3			J/J/J
*trnE*	6209/6253/6249	6273/6319/6317	65/67/69	1/14/2			J/J/J
*trnF*	6274/6318/6319	6341/6382/6383	68/65/65	–/−2/1			N/N/N
*nad5*	6342/6383/6384	8076/8123/8127	1735/1741/1744		ATT/ATT/ATT	T/T/T	N/N/N
*trnH*	8077/8124/8128	8145/8188/8192	69/65/65				N/N/N
*nad4*	8146/8189/8193	9493/9527/9531	1348/1339/1339		ATT/ATG/ATG	T/T/T	N/N/N
*nad4L*	9484/9534/9536	9771/9818/9820	288/285/285	−10/6/4	ATG/ATG/ATG	TAA/TAA/TAA	N/N/N
*trnT*	9781/9824/9826	9845/9887/9890	65/64/65	9/5/5			J/J/J
*trnP*	9846/9888/9891	9910/9952/9955	65/65/65				N/N/N
*nad6*	9913/9955/9958	10443/10485/10488	531/531/531	2/2/2	ATT/ATT/ATT	TAA/TAA/TAA	J/J/J
*cytb*	10446/10485/10491	11594/11636/11639	1149/1152/1149	2/−1/2	ATA/ATG/ATA	TAA/TAA/TAA	J/J/J
*trnS2*	11647/11635/11641	11714/11698/11705	68/64/65	52/−2/1			J/J/J
*nad1*	11733/11709/11728	12674/12659/12666	942/951/939	18/10/22	ATT/ATA/ATG	TAA/TAG/TAA	N/N/N
*trnL1*	12675/12663/12667	12743/12730/12734	69/68/68	–/3/–			N/N/N
*rrnL*	12739/12706/12794	14120/14082/14175	1382/1377/1382	−5/−25/59			N/N/N
*trnV*	14121/14083/14176	14185/14149/14240	65/67/65				N/N/N
*rrnS*	14185/14150/14241	14954/14917/15012	770/768/772	−1/–/–			N/N/N
NCR	14955/14918/15013	15333/15291/15381	379/374/369				J/J/J

Nucleotide composition of *A*. *virgata* is A = 39.7%, C = 11.8%, G = 7.5% and T = 41.0%. The base composition of *H*. *nephele* is A = 40.3%, C = 12.3%, G = 7.6% and T = 39.7%, and A = 39.8%, C = 12.2%, G = 7.7% and T = 40.2% in *O*. *maga*. The A + T content are 80.7%, 80.0% and 80.0%, respectively, showing a relatively strong A + T bias (Table 3). Compared with the whole‐genome, the noncoding A + T‐rich region (NCR) has the highest A + T content, up to 89.7%, 89.3%, and 91.9%, respectively. On the contrary, PCGs are the regions with the lowest A + T content, which is 79.1%, 78.4%, and 78.2% respectively. In addition, the T content of these mitogenomes on the major strand is higher than that of A, with the exception of *H*. *nephele* (Table 3).

**TABLE 3:** Nucleotide composition and skewness of different elements of mitogenomes of *Ampittia virgata*, *Halpe nephele*, and *Onryza maga*

Regions	Size (bp)	T(U)%	C%	A%	G%	A + T%	AT skew	GC skew
*A. virgata*/*H*. *nephele*/*O*. *maga*								
PCGs	11190/11202/11187	45.5/45.3/45.1	10.6/10.9/11.0	33.6/33.1/33.1	10.3/10.8/10.8	79.1/78.4/78.2	−0.150/−0.155/−0.153	−0.012/−0.002/−0.011
1st codon position	3730/3734/3729	37.3/37.5/37.0	10.2/10.3/10.6	37.3/36.5/36.7	15.2/15.7/15.7	74.6/74.0/73.7	0.000/−0.013/−0.005	0.196/0.208/0.191
2nd codon position	3730/3734/3729	48.2/48.2/48.1	16.4/16.6/16.7	22.3/22.3/22.0	13.2/13.0/13.2	70.5/70.5/70.1	−0.368/−0.367/−0.372	−0.108/−0.121/−0.117
3rd codon position	3730/3734/3729	51.0/50.1/50.2	5.1/5.7/5.7	41.3/40.4/40.7	2.6/3.7/3.	92.3/90.5/90.9	−0.105/−0.107/−0.105	−0.329/−0.207/−0.253
NCR	379/374/369	48.3/45.7/46.1	6.6/7.2/5.7	41.4/43.6/45.8	3.7/3.5/2.4	89.7/89.3/91.9	−0.076/−0.024/−0.003	−0.282/−0.350/−0.400
tRNAs	1458/1460/1457	39.6/40.3/40.1	7.7/7.5/7.8	42.0/41.4/41.2	10.7/10.7/10.9	81.6/81.7/81.3	0.029/0.014/0.014	0.164/0.173/0.165
rRNAs	2152/2145/2154	41.5/42.1/41.4	4.9/5.1/5.1	43.7/42.8/44.1	9.9/9.9/9.5	85.2/84.9/85.5	0.026/0.008/0.032	0.333/0.321/0.306
Full genome	15333/15291/15381	41.0/39.7/40.2	11.8/12.3/12.2	39.7/40.3/39.8	7.5/7.6/7.7	80.7/80.0/80.0	−0.017/0.007/−0.005	−0.220/−0.237/−0.222

### 3.2 Protein‐coding genes and codon usage

**FIGURE 2:**
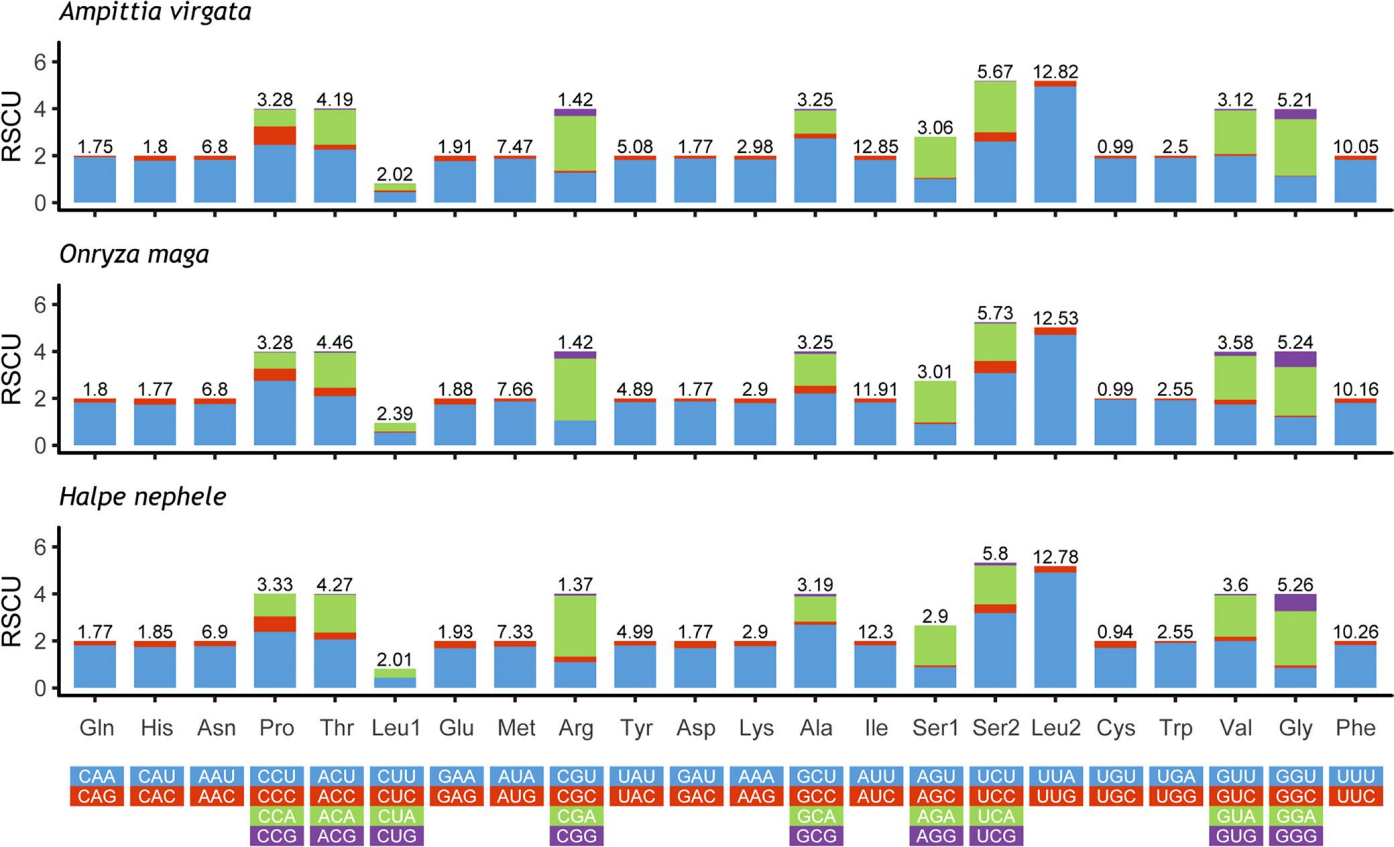
Relative synonymous codon usage (RSCU) in the mitogenomes of three Aeromachini species

The total lengths of the 13 PCGs of *Ampittia virgata*, *Halpe nephele,* and *Onryza maga* are 11,190 bp, 11,202 bp, and 11,187 bp, respectively (Table 3). In these 3 sequenced species, 9 of 13 PCGs (*nad2*, *cox1*, *cox2*, *atp8*, *atp6*, *cox3*, *nad3*, *nad6*, and *cytb*) are encoded in the J‐strand, and the other 4 (*nad5*, *nad4*, *nad4L*, and *nad1*) are located on the N‐strand. The size of the 13 PCGs with the smallest gene for the 13 PCGs is the *atp8,* and the largest gene is the *nad5* ranging in size from 162 bp to 1,744 bp. The AT‐skew and GC‐skew indicate that the T content of PCGs is obviously higher than that of A among these 3 species, while the content of G and C is not much different. The AT bias of the bases is more significant in the third codon, and the AT content of the third codon (90.5%–92.3%) is remarkably higher than that of the first codon (73.7%–74.6%) and the second codon (70.1%–70.5%), which is consistent with the higher mutation rate of the third codon site compared with the second and first codon sites (Table 3). All PCGs of these 3 mitogenomes start with typical ATN (ATG, ATT, and ATA) codons except *cox1* using CGA, and all of them use TAA or TAG as the stop codons, with the exception for *cox1*, *cox2*, *nad4,* and *nad5*, which use a single T as stop codons (Table 2). Statistics on the relative synonymous codon usage (RSCU) of the 3 skippers shows that the codon UUA (*Leu2*), UCU (*Ser2*), and CGA (*Arg*) are the 3 used most frequently, and the codons terminating with A and T also have a relatively higher frequency (Figure 2).

### 3.3 Transfer and ribosomal RNA genes

**FIGURE 3:**
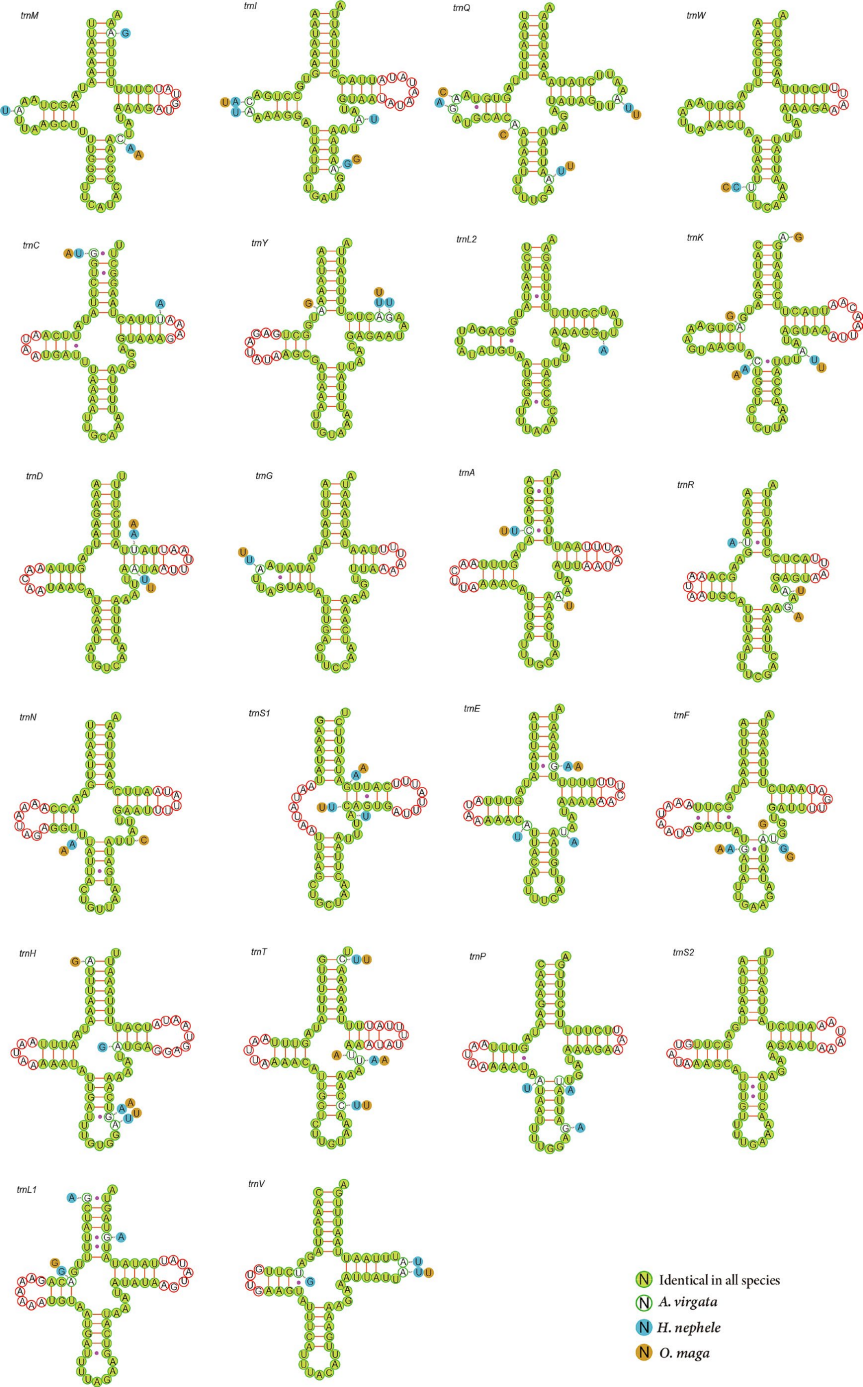
Predicted secondary clover‐leaf structure for the tRNA genes of *Ampittia virgata*, *Halpe nephele*, and *Onryza maga*

Each of the 3 skipper species harbor 22 tRNA genes, 14 of which are encoded in the J‐strand and 8 of them are encoded in the N‐strand, ranging from 63 bp to 69 bp in size (Table 2). The total lengths of the tRNA region of *A*. *virgata*, *H*. *nephele,* and *O*. *maga* are 1,458 bp, 1,460 bp, and 1,457 bp, respectively. The A + T content of tRNA is slightly higher than that of the PCGs (Table 3). Most tRNA genes of these 3 mitogenomes could be folded into a cloverleaf secondary structure, except for *trnS* (AGN), which lacks the DHU arm (Figure 3). The total number of unmatched base pairs found in the tRNAs of the 3 skippers was 28 in *O*. *maga*, 29 in *A*. *virgata,* and 34 in *H*. *nephele*. Most of these unmatched base pairs occur on the amino acid acceptor arm, the DHU arm and the anticodon arm, with only a few occurring on the TΨC arm. The majority of unmatched base pairs is U‐G which is a semi‐compensatory substitution; the others being U‐U A‐C, U‐C, A‐A, and A‐G mismatches (Figure 3).

The 2 rRNA genes (*rrnL* and *rrnS*) encoded by the N‐strand are located between *trnL* (CUN) and *trnV*, and between *trnV* and the A + T‐rich region, respectively. The large subunit rRNA (*rrnL*) is 1,382/1,377/1,382 bp (*A*. *virgata/H. nephele/O. maga*, respectively) in length while the small subunit rRNA (*rrnS*) is 770/768/772 bp (Table 2). In addition, both tRNA and rRNA of the three mitogenomes show a strong AT bias, which is higher than that of the whole mitogenomes (Table 3).

### 3.4 Overlapping sequences and intergenic spacers

There are 11, 8, and 5 gene overlapping regions in *A*. *virgata*, *H*. *nephele,* and *O*. *maga* mitogenomes, respectively, all ranging in size from 1 to 10 bp. The total lengths of the 3 mitogenomes ranges from 21 to 48 bp (Table 2). The longest of *A*. *virgata* mitogenomes is 10 bp located between *nad4* and *nad4L*, the longest of *H*. *nephele* is 25 bp located between *trnL1*‐*rrnL*, while the longest of *O*. *maga* is 8 bp located between *trnW‐trnC*. Four identical overlapping regions, namely the *nad2‐trnW* (2 bp), *trnW‐trnC* (8 bp), *atp8‐atp6* (7 bp), and *atp6‐cox3* (1 bp) are all present in these 3 mitogenomes (Table 2). Nineteen, thirteen, and sixteen intergenic spacers, ranging from 1 to 77 bp, from 2 bp to 53 bp and 1 bp to 78 bp, with their longest (77bp, 53bp, 78bp), are located between *trnQ* and *trnW*, are existed in *O*. *maga*, *A*. *virgata* and *H*. *nephele* mitogenomes, respectively (Table 2).

### 3.5 A + T‐rich region

**FIGURE 4:**
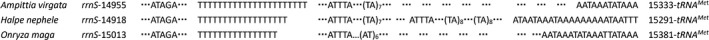
Structural element found in the AT‐rich region of 3 skippers (The presented nucleotides indicate the conserved sequences, Dots between sequences indicate omitted sequences)

The A + T‐rich region is the longest noncoding region with a relatively high A + T content, deemed to be related to the origin of replication and transcription (Boore, 1999; Cameron, 2014) and usually located between *rrnS* and *trnM*. In this study, this region ranges from 89.3% to 91.9%, with the longest (*A*. *virgata*) being 379 bp the second longest (*H*. *nephele*) being 374 bp, and the shortest (*O*. *maga*) being 369 bp in size (Table 3). In this study, a poly‐T and poly‐A stretches are all present with varying lengths in the A + T‐rich region. The poly‐T length ranges from 16 bp to 22 bp, and the poly‐A stretch ranges from 12 bp to 24 bp, often interrupted by the base T (Figure 4). These 2 types of T/A tandem repeats in the A + T‐rich region have been reported in other determined Hesperiidae mitogenomes (Han et al., 2018).

### 3.6 Phylogenetic relationships

**FIGURE 5:**
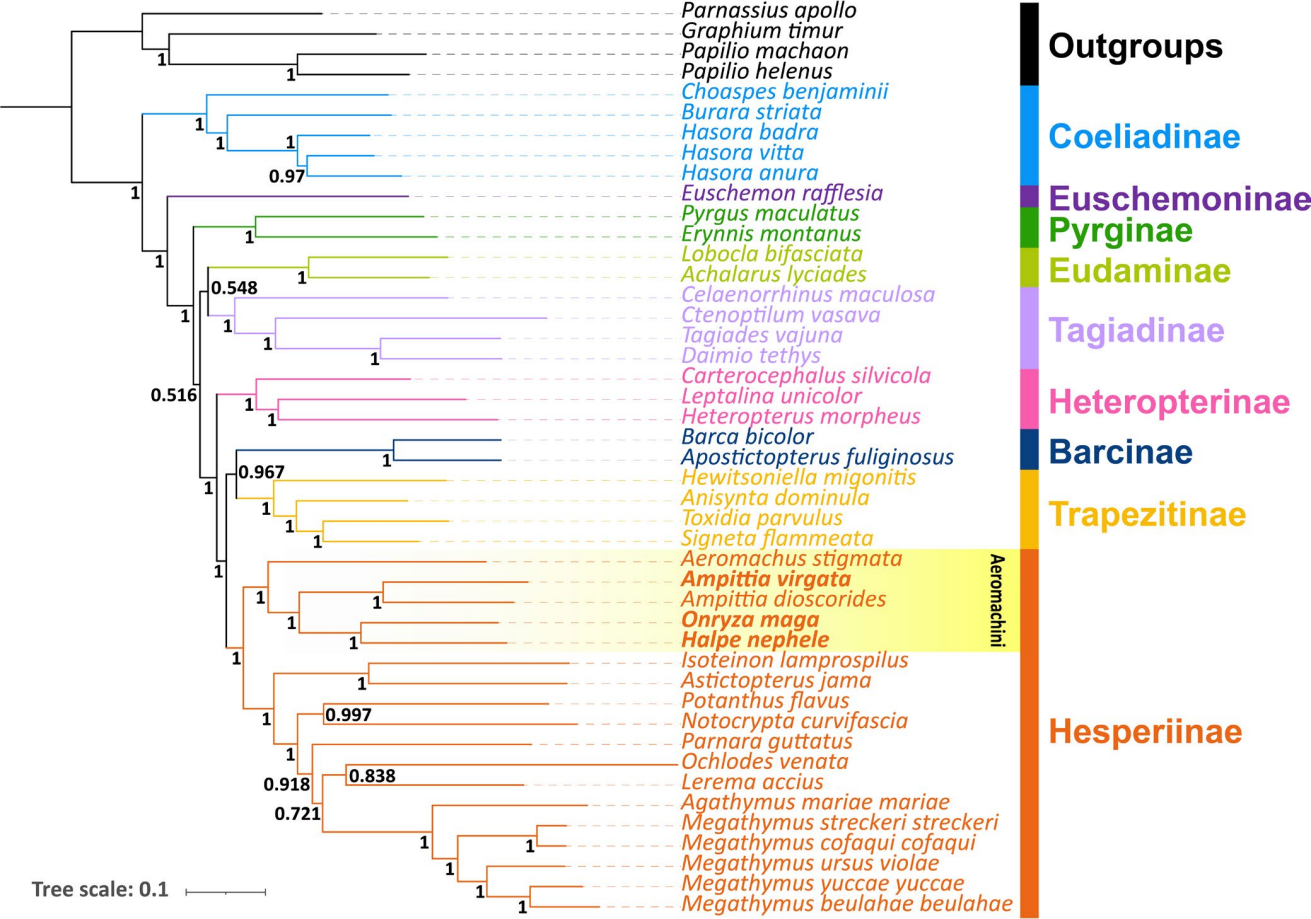
Phylogenetic tree inferred by BI method based on 12PRT dataset. Numbers on nodes are the posterior probabilities (PP)

In this study, we used the mitogenomes of a total of 45 hesperiid species containing 3 newly determined species (including 2 new mitogenomes in 2 genera) and 4 papilionid outgroup species to reconstruct the phylogenetic relationships of the tribe Aeromachini and closely related Hesperiinae in the family Hesperiidae. The analyses were conducted with ML and BI methods based on three datasets (PCGs, PRT and 12PRT). The results show that the obtained phylogenetic trees harbor almost the same topological structures, with nodes of the tree being strongly supported (the bootstrap support values, BS, of ML trees and the posterior probability, PP, of the BI trees). For simplicity and brevity, only one phylogenetic hypothesis (12PRT_BI) is presented here (Figure 5). The rest of the trees are in the Supplementary materials (Figure S1–S5).

The phylogenetic tree consists of 9 clades corresponding to 9 major hesperiid subfamilies sens Li, Cong, et al. (2019) and Zhang et al. (2019), and their relationships are Coeliadinae + (Euschemoninae + (Pyrginae + ((Eudaminae +Tagiadinae) + (Heteropterinae + ((Trapezitinae +Barcinae) + Hesperiinae))))) (Figure 5). The position of Eudaminae does not agree with that of previous studies where the subfamily is sister to the Pyrginae sensu lato, that is, Tagiadinae, Pyrrhopiginae, and Pyrginae sens Zhang et al. (2019). The inconsistency of phylogenetic relationships may be mainly caused by incomplete lineage sorting and inadequate taxon sampling (Pollard et al., 2006; Sahoo et al., 2016). In addition, Sahoo et al. (2016) thought the data from entire genomes may result in a better‐resolved phylogeny. On the contrary, Zhang et al. (2021) pointed out that phylogenetic trees based on whole‐genome sequence data may not always represent the true evolutionary history mainly due to the gene flow, which is also one possibility with appropriate references. However, it is difficult to effectively circumvent its influence firstly due to incomplete pedigree selection that results in long‐branch attraction and other phylogenetic errors and secondly due to the gene flow that causes the network evolution rather than the branched evolution. There are two taxonomic alternatives: (1) recognize three distinct subfamilies, Pyrginae, Eudaminae, and Tagiadinae as suggested by Zhang et al. (2019) of the subfamily Eudaminae is retained, or (2) combine them all as Pyrginae as in the conventional treatment. Here we tentatively adopt the former, pending further taxonomic investigation.

For the five Aeromachini species in this study, all results indicate that the Aeromachini form an independent clade in the subfamily Hesperiinae, which is the basal lineage among them (PP = 1), that *Aeromachus* branched at the root of the tribe, and that *H*. *nephele* and *O*. *maga* are sister groups (PP = 1), and the *H*. *nephele* + *O. maga* clade is sister to the *A*. *virgate* + *A*. *dioscorides* clade with strong support values (PP = 1). Huang et al. (2019) used two mitochondrial and two nuclear genes from 71 samples and Li, Zhu, et al. (2019) used 45 species in this tribe to infer phylogenetic relationships. Our results do not conflict with theirs. Although our analyses did not select sufficient samples of representative groups, for example, the Eudaminae, a satisfactorily clustering and high node support values were present in all the obtained trees.

*Apostictopterus fuliginosus* and *Barca bicolor* had been placed in the subfamily Heteropterinae in previous research (Warren et al., 2008, 2009; Yuan et al., 2015) until Han et al. (2018) proposed that *A*. *fuliginosus* and *B. bicolor* should be placed in subfamily Hesperiinae through phylogenetic analysis of mitogenomes. Subsequently, Zhang et al. (2019) adding Trapezitinae in their analysis, raised them to a subfamily rank Barcinae. Our results show that the two species and Trapezitinae are sister groups and the clade (*A*. *fuliginosus* + *B. bicolor* + Trapezitinae) is sister to the subfamily Hesperiinae with strong support values (PP = 1). Again, there are two taxonomic alternatives: (1) recognize 3 distinct subfamilies, Barcinae, Trapezitinae, and Hesperiinae as suggested by Li, Cong, et al. (2019), or (2) combine them all as Hesperiinae. Taxonomic treatment based only on molecular data is not desirable. Indeed, morphological synapomorphy is vague between Trapezitinae and Hesperiinae (Parsons, 1999; Warren et al., 2009). Here we refrain from drawing conclusion, pending further research.

## 4 CONCLUSIONS

In this study, we newly determined the mitogenomes of three hesperiid species in the tribe Aeromachini (*Ampittia virgata*, *Halpe nephele,* and *Onryza maga*) and provide more comprehensive molecular data for hesperiid phylogenetic study, meanwhile reconstructed the robust phylogenetic trees of hesperiid butterflies using relatively sufficient taxa sampling based on multiple mitogenomic datasets. The size and structure of mitochondria, gene order, and AT content of these three species are highly consistent with other Lepidoptera species. The phylogenetic analysis results show that Aeromachini is a monophyletic group and sister to the rest of Hesperiinae and that the relationships among hesperiid subfamilies are Coeliadinae + (Euschemoninae + (Pyrginae + ((Eudaminae + Tagiadinae) + (Heteropterinae + ((Trapezitinae + Barcinae) + Hesperiinae))))). Moreover, our analysis supports the viewpoint of previous study that *A*. *fuliginosus* and *B. bicolor* should be placed out of the subfamily Hesperiinae.

## CONFLICTS OF INTEREST

All authors report no conflicts of interest.

## AUTHOR CONTRIBUTIONS

**Xiangyu Hao:** Conceptualization (equal); Software (equal); Validation (equal); Writing‐original draft (lead). **Jiaqi Liu:** Methodology (equal); Software (equal). **Hideyuki Chiba:** Conceptualization (equal); Methodology (equal); Writing‐review & editing (lead). **Jintian Xiao:** Methodology (equal); Software (equal). **Xiangqun Yuan:** Conceptualization (equal); Funding acquisition (lead); Project administration (lead); Supervision (lead); Validation (equal); Writing‐review & editing (supporting).

## Supporting information

Supplementary Material

## Data Availability

The following information was supplied regarding the availability of DNA sequences: The complete mitogenomes of *Ampittia virgata*, *Halpe nephele,* and *Onryza maga* are deposited in GenBank of NCBI under accession number MW288057, MW288058, and MW288059, respectively.
