# Supplementary material for: Complete mitochondrial genomes of three skippers in the tribe Aeromachini (Lepidoptera: Hesperiidae: Hesperiinae) and their phylogenetic implications
Source: Ecol Evol. 2021 May 18;11(12):8381–93. doi: 10.1002/ece3.7666 (PMC8216930; doi:10.1002/ece3.7666)
Supplement: Supplementary file 1 — Supplementary Material [file ECE3-11-8381-s001.docx]

Supplementary Materials

Three Complete Mitochondrial Genomes of the Tribe Aeromachini (Lepidoptera: Hesperiidae: Hesperiinae) and Their Phylogenetic Implications

Xiangyu Hao ^1^, Jiaqi Liu ^2^, Hideyuki Chiba^3^, Jintian Xiao^2^ and Xiangqun Yuan ^2,^*

^1^ College of Life Sciences, Northwest A&F University, Yangling, Shaanxi 712100, China; [xyhao@nwsuaf.edu.cn](mailto:xyhao@nwsuaf.edu.cn)

^2^ Key Laboratory of Plant Protection Resources and Pest Management, Ministry of Education, Entomological Museum, College of Plant Protection, Northwest A&F University, Yangling, Shaanxi 712100, China; [jiaq_work@163.com](mailto:jiaq_work@163.com) (J.L.); [xjt0629@nwafu.edu.cn](mailto:xjt0629@nwafu.edu.cn) (J.X.)

^3^ B.P. Bishop Museum, Honolulu, Hawaii 96817-0916, USA; [chiba.zootaxa@gmail.com](mailto:chiba.zootaxa@gmail.com)

***** Correspondence: [yuanxq@nwsuaf.edu.cn](mailto:yuanxq@nwsuaf.edu.cn); Tel.: +86-1375-998-5152

**Table S1.** Best partitioning schemes and models based on different datasets for BI analysis.

| **Dataset** | **Partitioning scheme** | **Models** |
| --- | --- | --- |
| PCGs | P1: (*cox3*_ pos1, *atp*6_ pos1, *cytb*_pos1) | GTR+I+G |
|  | P2: (*cox1*_pos2, *atp6*_pos2, *nad3*_pos2, *cox2*_pos2, *cox3*_pos2, *cytb*_pos2) | GTR+I+G |
|  | P3: (*atp6*_pos3, *cox2*_pos3, *nad6*_pos3, *atp8*_pos3, *nad2*_pos3, *cox1*_pos3) | GTR+G |
|  | P4: (*atp8*_pos1, *nad6*_pos1, *nad2*_pos1, *nad3*_pos1) | GTR+I+G |
|  | P5: (*nad2*_pos2, *atp8*_pos2, *nad6*_pos2) | GTR+I+G |
|  | P6: (*cox2*_pos1, *cox1*_pos1) | GTR+I+G |
|  | P7: (*nad3*_pos3, *cox3*_pos3, *cytb*_pos3) | GTR+I+G |
|  | P8: (*nad5*_pos1, *nad1*_pos1, *nad4*_poss1, *nad4L*_pos1) | GTR+I+G |
|  | P9: (*nad4*_pos2, *nad4L*_pos2, *nad5*_pos2, *nad1*_pos2) | GTR+I+G |
|  | P10: (*nad1*_pos3, *nad4*_pos3) | GTR+G |
|  | P11: (*nad4L*_pos3, *nad5*_pos3) | GTR+I+G |
| PRT | P1: (*trnL2*, *cox3*_pos1, *atp6*_pos1, *cytb*_pos1) | GTR+I+G |
|  | P2: (*atp6*_pos2, *cox1*_pos2, *cox2*_pos2, *cox3*_pos2, *cytb*_pos2) | GTR+I+G |
|  | P3: (*nad2*_pos3, *atp8*_pos3, *cox2*_pos3, *cox1*_pos3, a*tp6*_pos3) | GTR+G |
|  | P4: (*nad3*_pos1, *nad2*_pos1, *trnS1*, *trnA*, *trnT*, *nad6*_pos1, *atp8*_pos1) | GTR+I+G |
|  | P5: (*trnE*, *trnR*, *atp8*_pos2) | F81+I+G |
|  | P6: (*cox2*_pos1, *cox1*_pos1) | GTR+I+G |
|  | P7: (*nad6*_pos3, *nad3*_pos3, *cox3*_pos3, *cytb*_pos3) | GTR+G |
|  | P8: (*trnY*, *nad5*_pos1, *nad1*_pos1, *nad4*_pos1, *nad4L*_pos1) | GTR+I+G |
|  | P9: (*nad4*_pos2, *nad4L*_pos2, *nad5*_pos2, *nad1*_pos2) | GTR+I+G |
|  | P10: (*nad1*_pos3, *nad4*_pos3) | GTR+G |
|  | P11: (*nad2*_pos2, *nad3*_pos2, *nad6*_pos2) | GTR+I+G |
|  | P12: (*nad4L*_pos3, *nad5*_pos3) | GTR+I+G |
|  | P13: (*trnP*, *rrnL*, *trnV*, *trnN*, *rrnS*, *trnI*) | GTR+I+G |
|  | P14: (*trnM*, *trnW*, *trnH*, *trnC*, *trnK, trnO*, *trnF*, *trnL1*) | HKY+I+G |
|  | P15: (*trnG*, *trnS2*, *trnD*) | F81+I+G |
| 12PRT | P1: (*atp6*, *nad3*, *cox1*, *cox3*, *trnE*, *cox2*, *cytb*) | GTR+I+G |
|  | P2: (*nad6*, *atp8*) | GTR+I+G |
|  | P3: (*nad4*, *nad4L*, *nad5*, *nad1*) | GTR+I+G |
|  | P4: (*nad2*) | GTR+I+G |
|  | P5: (*trnY*, *trnP*, *rrnL*, *trnV*, *trnH*, *trnN*, *rrnS*, *trnI*) | GTR+I+G |
|  | P6: (*trnL2*, *trnS1*, *trnT*, *trnA*, *trnG*, *trnS2*, *trnD*, *trnR*) | GTR+I+G |
|  | P7: (*trnM*, *trnW*, *trnL1*, *trnF*, *trnQ*, *trnC*, *trnK*) | HKY+I+G |

**Table S2.** Best partitioning schemes and models based on different datasets for ML analysis.

| **Datasets** | **Partitioning scheme** | **Models** |
| --- | --- | --- |
| PCGs | P1: (*cox3*_pos1, *cytb*_pos1, *atp6*_pos1) | GTR+I+G |
|  | P2: (*atp6*_pos2, *cox1*_pos2, *cox3*_pos2, *cytb*_pos2, *ztp6*_pos2) | TVM+I+G |
|  | P3: (*nad6*_pos3, *atp8*_pos3, *cox2*_pos3, *atp6*_pos3, *nad2*_pos3, *cox1*_pos3) | TIM+G |
|  | P4: (*nad3*_pos1, *nad2*_pos1, *nad6*_pos1, *atp8*_pos1, *atp8*_pos1) | GTR+I+G |
|  | P5: (*cox2*_pos1, *cox1*_pos1) | GTR+I+G |
|  | P6: (*nad3*_pos3, *cox3*_pos3, *cytb*_pos3) | TIM+I+G |
|  | P7: (*nad5*_pos1, *nad1*_pos1, *nad4L*_pos1, *nad4*_pos1) | GTR+I+G |
|  | P8: (*nad4L*_pos2, *nad4*_pos2, *nad5*_pos2, *nad1*_pos2) | GTR+I+G |
|  | P9: (*nad1*_pos3, *nad4*_pos3) | GTR+G |
|  | P10: (*nad6*_pos2, *nad3*_pos2, *nad2*_pos2) | TVM+I+G |
|  | P11: (*nad4L*_pos3, *nad5*_pos3) | K81UF+I+G |
| PRT | P1: (*trnL2*, *cox3*_pos1, *cytb*_pos1, *atp6*_pos1) | GTR+I+G |
|  | P2: (*atp6*_pos2, *cox1*_pos2, *cox3*_pos2, *cytb*_pos2, *cox2*_pos2) | TVM+I+G |
|  | P3: (*nad6*_pos3, *atp8*_pos3, *cox2*_pos3, a*tp6*_pos3, *atp6*_pos3) | TRN+G |
|  | P4: (*nad2*_pos1, *nad3*_pos1, *trnS1*, *trnA*, *trnT*, *nad6*_pos1, *atp8*_pos1, *trnE*, *atp8*_pos2) | GTR+I+G |
|  | P5: (*cox2*_pos1, *cox1*_pos1) | GTR+I+G |
|  | P6: (*nad2*_pos3, cox1_pos3) | TIM+G |
|  | P7: (*nad3*_pos3, *cox3*_pos3, *cytb*_pos3) | TIM+I+G |
|  | P8: (*nad5*_pos1, *nad1*_pos1, *trnY*, *nad4L*_pos1, *nad4*_pos1) | GTR+I+G |
|  | P9: (*nad4L*_pos2, *nad4*_pos2, *nad5*_pos2, *nad1*_pos2) | GTR+I+G |
|  | P10: (*nad1*_pos3, *nad4*_pos3) | GTR+G |
|  | P11: (*nad3*_pos2, *nad2*_pos2, *trnK*, *trnC*, *nad6*_pos2) | TVM+I+G |
|  | P12: (*nad4L*_pos3, *nad5*_pos3) | TIM+I+G |
|  | P13: (*trnP*, *rrnL*, *trnV*, *rrnS*, *trnR*, *trnN*, *trnI*) | GTR+I+G |
|  | P14: (*trnG*, *trnD*, *trnS2*) | F81+I+G |
|  | P15: (*trnG*, *trnD*, *trnS2*) | TRN+I+G |
| 12PRT | P1: (*atp6*, *nad3*, *trnS1*, *trnT*, *trnA*) | GTR+I+G |
|  | P2: (*nad6*, *atp8*) | GTR+I+G |
|  | P3: (*cox3*, *cox1*, *trnE*, *cox2*, *cytb*) | GTR+I+G |
|  | P4: (*nad1*, *nad5*, *nad4L*, *nad4*) | GTR+I+G |
|  | P5: (*nad2*) | TIM+I+G |
|  | P6: (*trnY*, *trnP*, *rrnL*, *trnV*, *trnH*, *rrnS*, *trnR*, *trnI*, *trnN*) | GTR+I+G |
|  | P7: (*trnM*, *trnW*, *trnK*, *trnC*, *trnL2*, *trnF*, *trnQ*, *trnL1*) | HKY+I+G |
|  | P8: (*trnG*, *trnD*, *trnS2*) | F81+I+G |


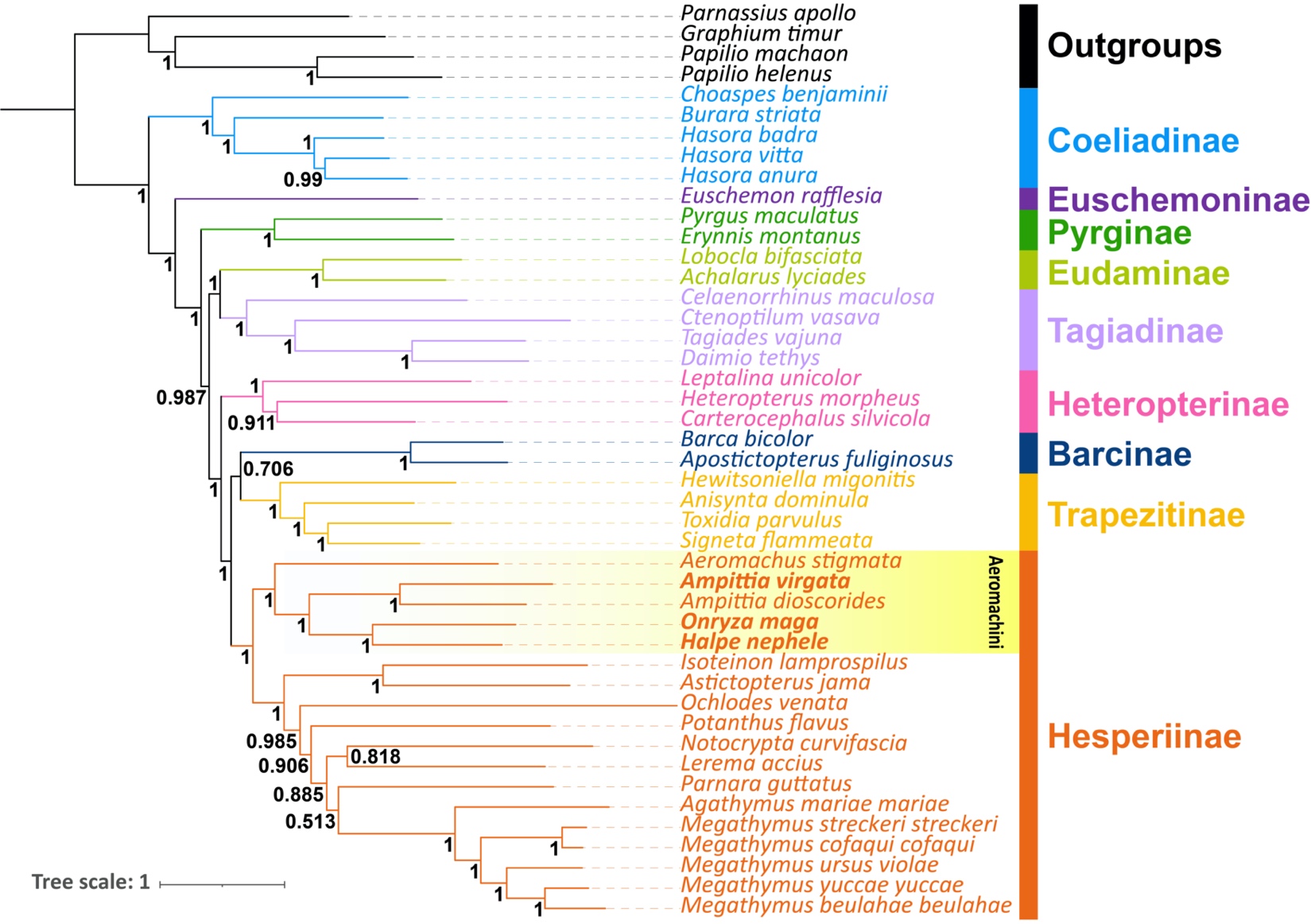


**Figure S1.** Phylogenetic tree inferred by BI method based on PCGs dataset. Numbers on nodes are the posterior probabilities (PP).


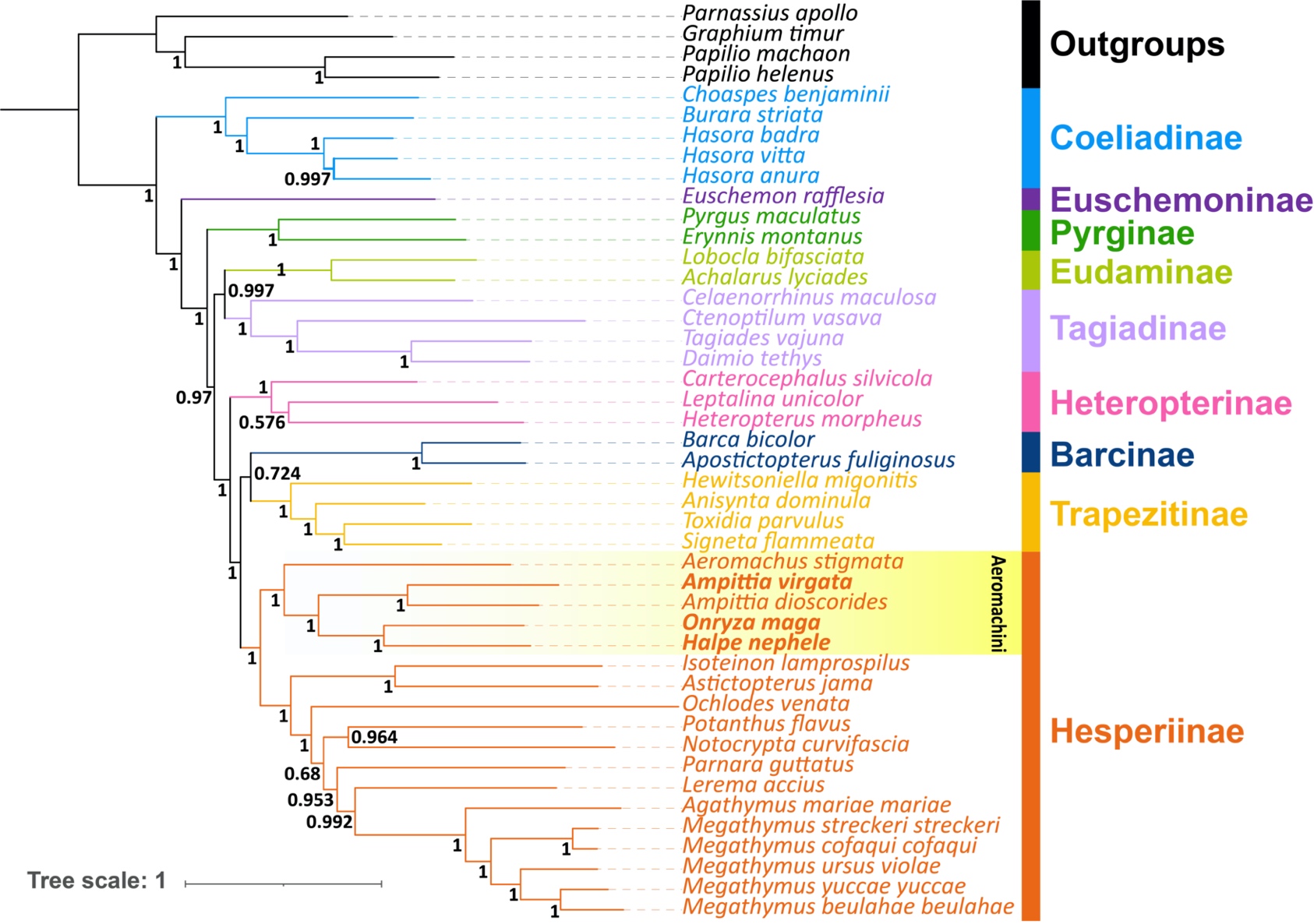


**Figure S2.** Phylogenetic tree inferred by BI method based on PRT dataset. Numbers on nodes are the posterior probabilities (PP).


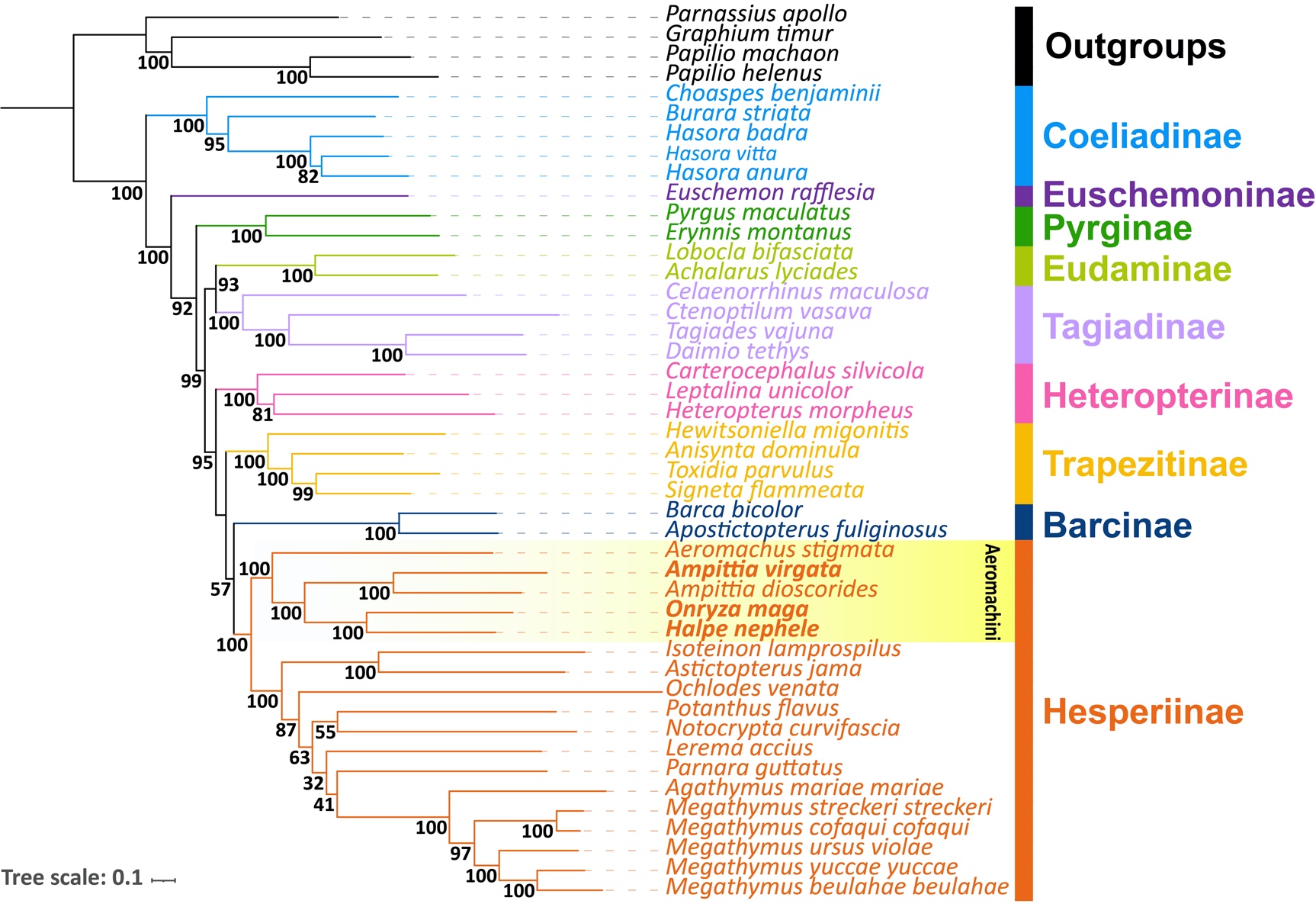


**Figure S3.** Phylogenetic tree inferred by ML method based on PCGs dataset. Numbers on nodes are the bootstrap support values (BS).


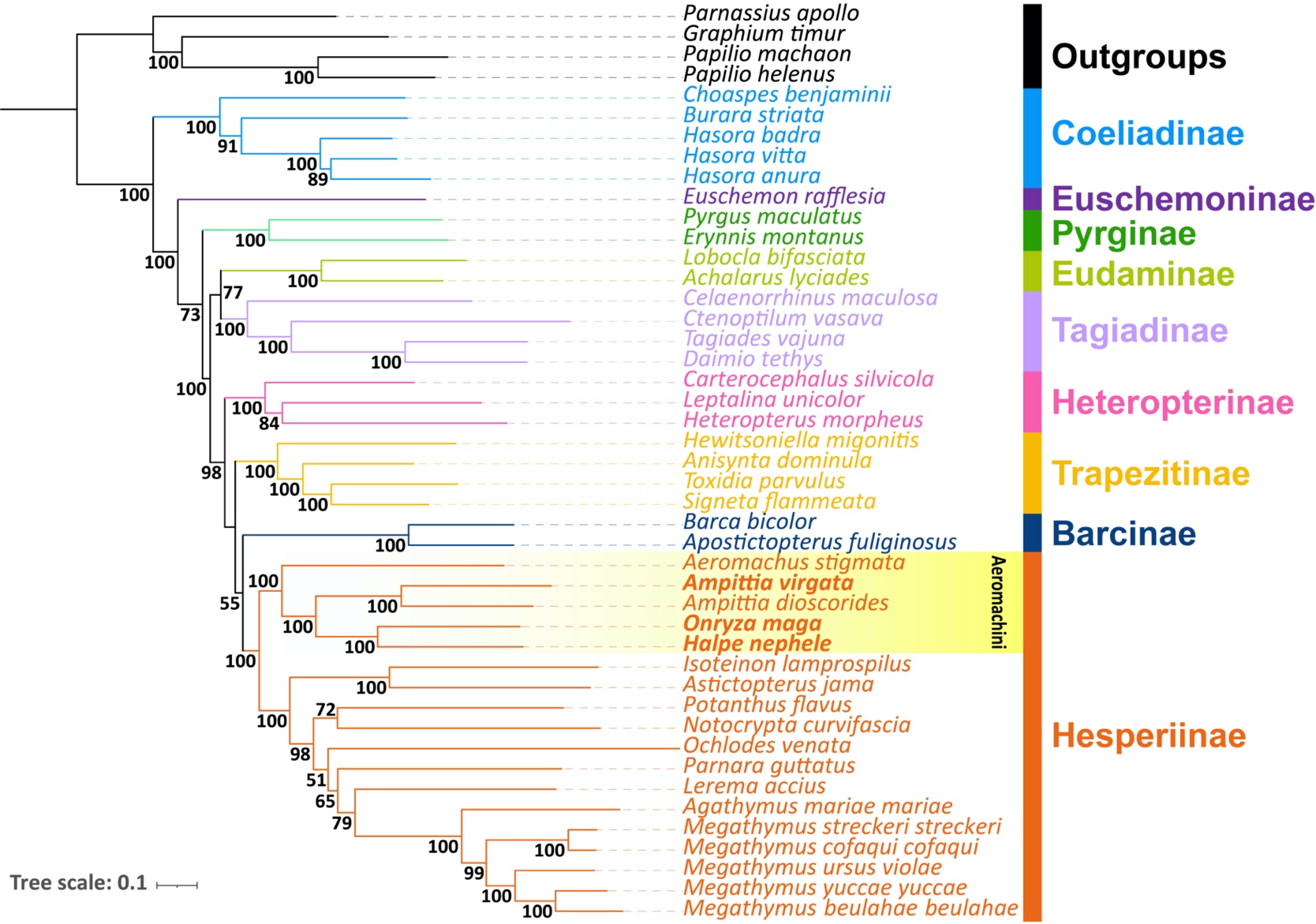


**Figure S4.** Phylogenetic tree inferred by ML method based on PRT dataset. Numbers on nodes are the bootstrap support values (BS).


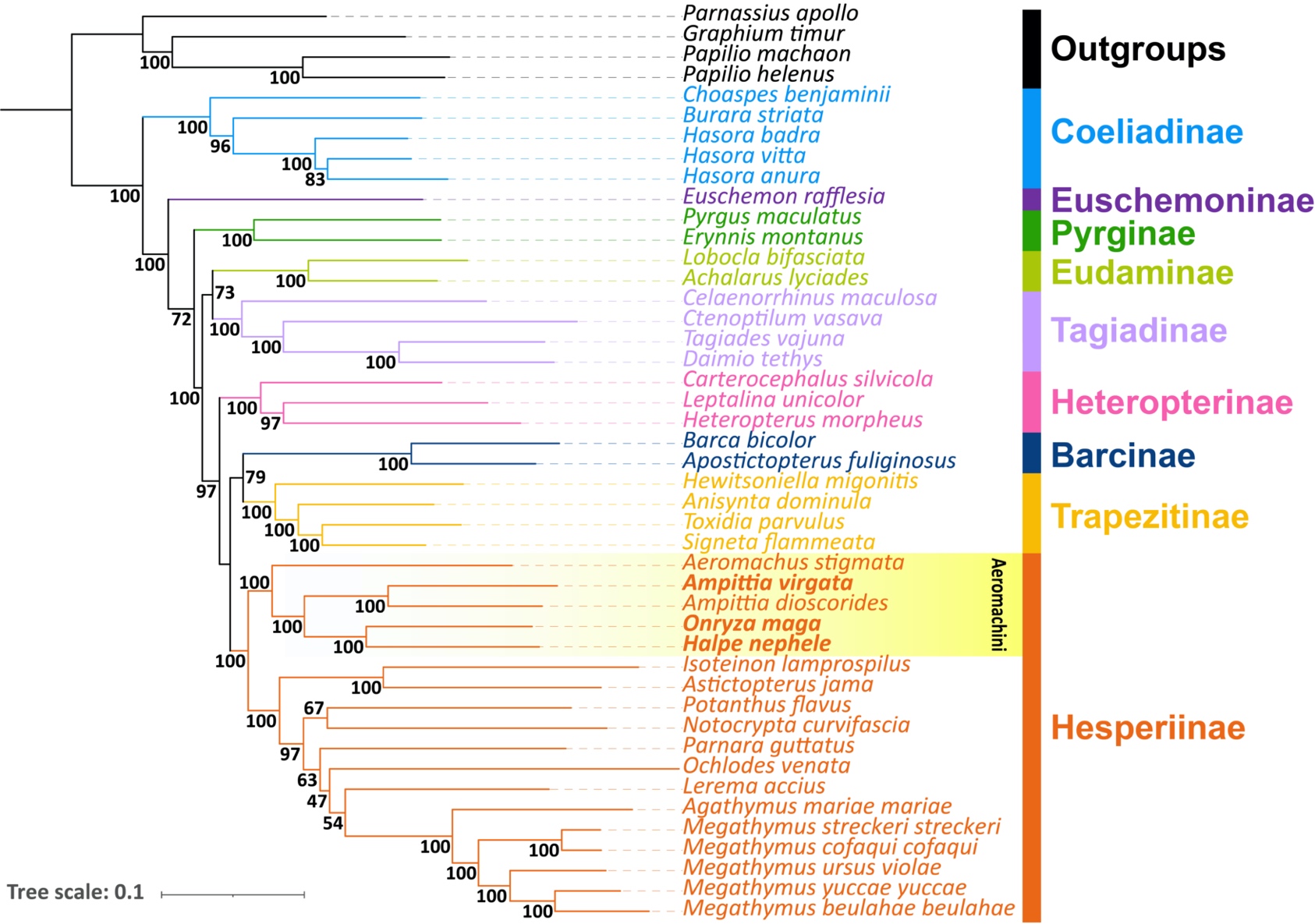


**Figure S5.** Phylogenetic tree inferred by ML method based on 12PRT dataset. Numbers on nodes are the bootstrap support values (BS).
